# An Unbiased Drug Screen in a Drosophila Model of *LMNA*-Muscular Dystrophy Identifies Calcium Channel Blockers as Potential Treatments

**DOI:** 10.3390/ijms27188367

**Published:** 2026-09-19

**Authors:** Nathaniel P. Mohar, Brenna A. Powers, Benjamin E. Hinz, Alex R. Bock, Hailey McCoy-Munger, Zachary Darr, Maxwell W. Shumaker, Alexander T. Wemmie, Amya Saxena, Lori L. Wallrath

**Affiliations:** 1 Interdisciplinary Graduate Program in Genetics, University of Iowa, Iowa City, IA 52242, USA; nathaniel-mohar@uiowa.edu; 2 Department of Biochemistry and Molecular Biology, Carver College of Medicine, University of Iowa, Iowa City, IA 52242, USA; brenna-powers@uiowa.edu (B.A.P.); hailey-mccoy-munger@uiowa.edu (H.M.-M.); maxwell-shumaker@uiowa.edu (M.W.S.); sasha-wemmie@uiowa.edu (A.T.W.); amya-saxena@uiowa.edu (A.S.); 3 Department of Biomedical Engineering, University of Iowa, Iowa City, IA 52242, USA

**Keywords:** calcium channels, *Drosophila melanogaster*, drug repurposing, Emery–Dreifuss muscular dystrophy, lamins, laminopathies

## Abstract

Mutations in the *LMNA* gene encoding A-type lamins cause multiple muscular dystrophies (*LMNA*-MD). Lamins are intermediate filaments with three conserved domains: an N-terminal head, a coiled-coil rod, and a C-terminal domain possessing an Ig-like fold. Missense mutations in all three domains can cause *LMNA*-MD by mechanisms that are not well understood. Currently, there are limited treatments for *LMNA*-MD beyond symptom management. In this study, our goal was to identify candidate treatments through an in vivo drug repurposing screen using Drosophila models of *LMNA*-MD that recapitulate aspects of the disease phenotype. Expression of *LamC* R264Q (Drosophila orthologue of human *LMNA* R249Q) in fly muscles causes muscle defects and premature death. We fed 1520, mostly FDA/EMA-approved, drugs to larvae and screened for rescue of lethality. This screen resulted in 68 positive hit drugs that partially restored viability. Molecular fingerprinting clustered a subset of these compounds into six structurally related groups, including L-type voltage-gated calcium channel inhibitors. We tested these inhibitors using multiple fly models expressing different mutant lamins and discovered that only flies expressing amino acid substitutions in the rod domain benefited, supporting a need for personalized treatments. Collectively, our findings support L-type voltage-gated calcium channel blockers as candidate treatments that warrant testing in pre-clinical models.

## 1. Introduction

Lamins are intermediate filaments that form a meshwork underlying the inner nuclear membrane (INM) known as the nuclear lamina [1]. Lamins are subdivided into the B-type and A-type, with the B-type lamins B1 and B2 encoded by the *LMNB1* and *LMNB2* genes, respectively, and the A-type lamins A and C encoded by the *LMNA* gene via alternative splicing [1,2]. All lamins have three conserved structural regions: a globular N-terminal head, an alpha-helical central rod, and a C-terminal tail, which includes a nuclear localization sequence and an Ig-like fold domain. Lamins homopolymerize to form filaments. This process begins with the formation of lamin dimers, which result from the formation of coiled-coils via interactions between the alpha-helices of the central rod. These dimers combine to form head-to-tail protofilaments, which then interact in an anti-parallel lateral manner through the coil 1b and coil 2 regions of the rod to form the higher-order structure of the lamina. Super-resolution microscopy revealed that A- and B-type lamins form independent networks. Prior to cellular differentiation, B-type lamins form a meshwork underlying the inner nuclear membrane. As cells differentiate, A-type lamins are expressed and form a second meshwork between the B-type lamins and the nucleoplasm [3].

Mutations in lamin-encoding genes cause a collection of human diseases known as laminopathies. While there are diseases associated with *LMNB1* and *LMNB2* mutations, the vast majority of laminopathies are caused by mutations in the *LMNA* gene [4,5,6,7]. There are at least 16 diseases associated with approximately 500 pathogenic mutations across the full length of the *LMNA* gene, with the majority of these inherited in an autosomal dominant fashion [6,7,8,9,10,11,12]. Phenotypes associated with *LMNA* mutations can be broadly divided into accelerated aging syndromes, diseases affecting adipose tissue, peripheral neuropathies, and striated muscle diseases, which are the most common and account for 55–79% of laminopathy cases [13,14]. In addition, there is poor genotype–phenotype correlation, making disease symptoms difficult to predict [6,7,8,9,10,11,12,13,14].

The striated muscle laminopathies are Emery–Dreifuss muscular dystrophy (EDMD), limb girdle muscular dystrophy type 1B (LGMD1B), lamin-associated congenital muscular dystrophy (L-CMD), and dilated cardiomyopathy with conduction defects (DCM-CD) [15,16,17,18]. Muscular dystrophies caused by *LMNA* mutations are collectively referred to as *LMNA*-MD, with phenotypes existing on a continuous spectrum where CMD is the most severe phenotype [15,19]. DCM-CD can either present without skeletal muscle involvement or co-occur with *LMNA*-MD [18,19]. The overwhelming majority of striated muscle laminopathies are caused by dominantly inherited missense mutations in *LMNA* that affect all domains of the lamin A/C protein [7,20,21]. This includes the rod domain, where commonly occurring substitutions include H222P and R249Q, often associated with EDMD, and R249W, often associated with L-CMD [20,21,22,23].

There are currently limited treatments for *LMNA*-MD other than symptom management [1,24]. However, there is an ongoing clinical trial for *LMNA*-associated DCM testing NVC-001 (Nuevocor Pte. Ltd., Singapore), an adeno-associated virus encoding a truncated version of the nuclear envelope protein SUN1 [25]. Treatments used for other muscular dystrophies often have limited relevance to *LMNA*-MD. The standard of care pharmacological treatment for Duchenne muscular dystrophy (DMD) is corticosteroids [26,27]. Reports on the effectiveness of corticosteroids for *LMNA*-MD are variable and largely restricted to L-CMD [28,29,30]. Gene therapies, which include splice-modifying antisense oligonucleotides (ASOs) and viral vector gene replacement, are cutting-edge treatments with significant success for other types of muscular dystrophies [31,32,33,34,35]. Limited development of splice-modifying ASOs for *LMNA*-MD has occurred, with efficient skipping demonstrated only for *LMNA* exon 5 in human cells [36]. Viral vector gene replacement in its current form is unlikely to be successful for *LMNA*-MD, as it is currently only used to treat diseases caused by loss-of-function mutations [31,33,34,35]. Mutations that cause *LMNA*-MD are typically dominantly inherited and encode a protein with a single amino acid substitution that acts as a dominant negative, minimizing the therapeutic benefit of overexpressing a wild-type protein [7,23,29,37,38]. Additionally, there are significant safety and economic concerns surrounding viral vector gene therapies [39,40,41]. Thus, while gene therapy development remains a long-term goal, pharmacological approaches offer a faster route to translation for dominantly inherited diseases caused by pathogenic missense mutations.

Muscular dystrophy has been extensively modeled in the fruit fly *Drosophila melanogaster* [42]. DMD, LGMD, and EDMD have been successfully modeled in Drosophila, with phenotypes resembling the progressive loss of muscle function, locomotor ability, and cardiac function as seen in human patients [20,43,44,45,46,47,48,49,50,51,52,53,54,55,56,57,58,59,60,61]. Drosophila models of human diseases have been used in drug screens with the goal of identifying human disease mechanisms and potential treatments [62,63]. Suppression of lethality is often used as a readout in these screens, as feeding drugs to Drosophila larvae can result in easily scorable rescue of pupal or adult viability, leading to relatively high-throughput screening [62,63]. There are multiple examples of this approach in the oncology space [62,63,64,65,66,67], as well as in Drosophila models of congenital glycosylation disorders [68,69]. However, to our knowledge, a drug repurposing screen has yet to be completed in a Drosophila model of muscular dystrophy.

To address the limited treatments currently available for *LMNA*-MD, we completed an unbiased drug screen using the Prestwick Chemical Library to rescue lethality caused by expression of mutant A-type lamins. Drosophila Lamin C (LamC) R264Q is orthologous to human lamin A/C R249Q, a substitution which most commonly causes EDMD [20]. Expression of a *LamC* R264Q transgene exclusively in larval body wall muscles causes lethality at the pupal stage [20]. This screen identified 68 drugs that rescued adult viability. Clustering of these 68 hit compounds by molecular structure identified six classes with related structures. We followed up on L-type voltage-gated calcium channel blockers given the physiological relevance of calcium signaling to muscle function, including mouse models of *Lmna*-associated cardiac disease [70,71,72,73]. We found that multiple classes of L-type calcium channel blockers rescue the lethality caused by muscle-specific expression of specific mutant lamins. Collectively, our findings demonstrate the utility of Drosophila models as powerful tools for in vivo screening, support L-type calcium channel antagonists as potential drug repurposing candidates and suggest the need for personalized treatments for *LMNA*-MD.

## 2. Results

### 2.1. A Drug Screen Was Performed for Rescue of Lethality Caused by LamC R264Q

**Figure 1:**
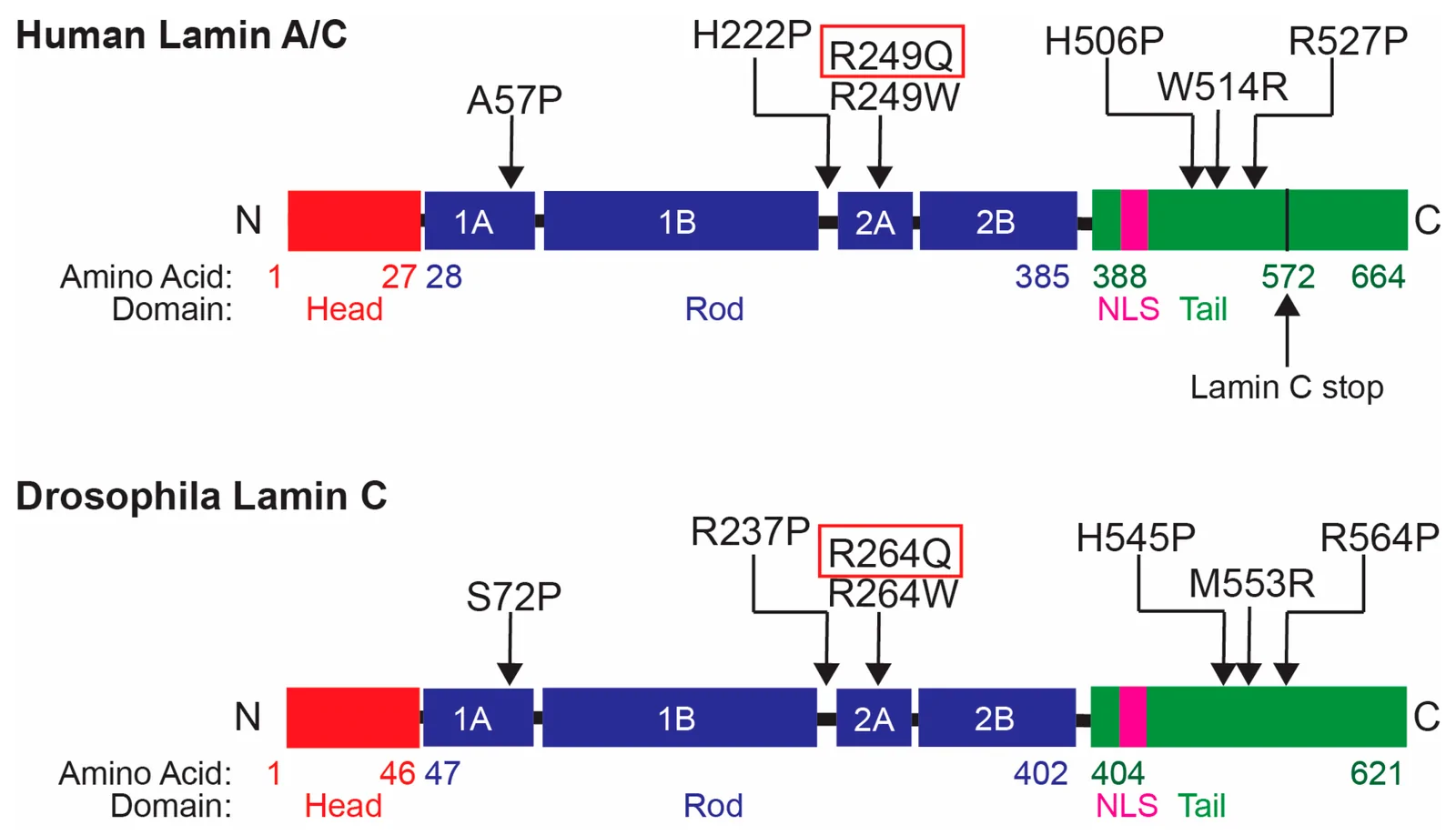
Schematic of the human and Drosophila A-type lamin structure showing the amino acid substitutions studied. The primary drug screen was completed using a Drosophila model of LamC R264Q that corresponds to human lamin A/C R249Q. Drosophila models of the additional amino acid substitutions were used for subsequent drug tests. Each substitution in Drosophila LamC is orthologous to a known pathogenic substitution in human lamin A/C (positions indicated by arrows).

There are few treatments available for *LMNA*-MD, with standard care consisting of symptom management without treating the cause of the disease [30]. We have previously developed and characterized a Drosophila model transgenic for *LamC* R264Q [20]. The R264Q substitution in Drosophila LamC is orthologous to the human lamin A/C R249Q substitution (Figure 1). This amino acid substitution affects the rod domain of the A-type lamin protein and is primarily associated with EDMD in humans [23]. Expression of *LamC* R264Q in the Drosophila larval body wall muscles results in completely penetrant lethality at the pupal stage [20]. Lethality is accompanied by reduced A- and B-type lamins at the nuclear periphery, perinuclear aggregation of A-type lamins, and nucleoplasmic aggregation of B-type lamins and nuclear pores, resulting in nuclear lobulations [20]. Taken together, these findings demonstrate that this single amino acid substitution in the rod domain of A-type lamins can alter nuclear architecture in intact muscle within a developing organism [20].

The binary nature of the complete loss of adult viability caused by *LamC* R264Q expression represents an optimal phenotype for drug screening. To identify novel disease mechanisms and treatments for *LMNA*-MD, we completed an unbiased screen of the Prestwick Chemical Library (version 20, Prestwick Chemical, Illkirch, France) for rescue of lethality caused by expression of *LamC* R264Q in the larval body wall muscles of Drosophila. The Prestwick Chemical Library contains 1520 off-patent drugs with over 600 known biological targets. Ninety-eight percent of drugs in the library are either FDA- or EMA-approved and are known to be safe in humans, allowing the library to be used in disease models to identify drug repurposing candidates. The library has previously been used for lethality suppression screens in Drosophila to identify candidate treatments for other genetic diseases, making it ideal for our application [68,69].

**Figure 2:**
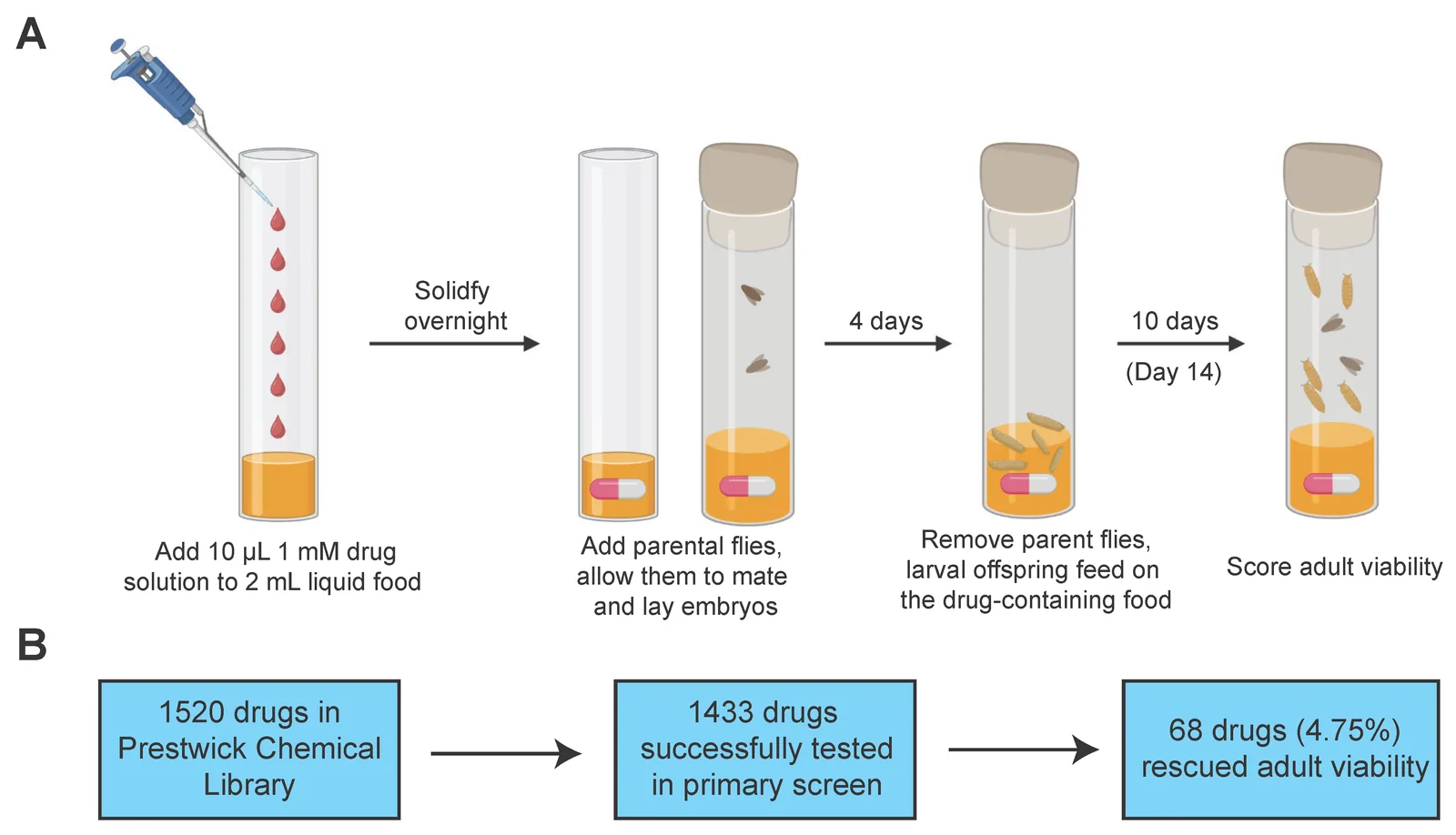
Methods and results of the primary screen. (**A**) Schematic representation of the primary screening procedure. (**B**) A flowchart of the results of the primary screen indicating that 68 drugs rescued adult viability following expression of *LamC* R264Q.

The Prestwick Chemical Library was purchased with each drug pre-dissolved at 10 mM in DMSO. For our screen, these drugs were diluted to 1 mM and 10% DMSO in PBS. Each drug was mixed with 2 mL of liquid fly food per vial, which resulted in a final concentration of 5 µM drug and 0.05% DMSO. For the primary screen, each drug was tested in a single vial at a single concentration of 5 µM. Thus, we recognize that our results could be an underestimation of the number of positive compounds. Parental flies possessing the *C57*-Gal4 driver were mated to flies possessing the *LamC* R264Q transgene in each vial. After mating, 100% of the offspring produced by these parents express the *LamC* R264Q transgene exclusively in larval body wall muscles [74,75]. The parental generation was removed from the vials, and larval progeny consumed the food containing either DMSO alone or the drug throughout the larval stages, until they pupated on the walls of the vials. After 14 days, adult viability was scored (Figure 2A). Given the complete penetrance of lethality in the absence of drug treatment (food alone or food plus DMSO only) [20], the emergence of even a single adult fly out of ~30 pupae per vial following drug treatment was considered a positive hit in the primary screen.

To validate this approach, we first tested whether DMSO alone altered viability. We treated 902 total progeny expressing a wild-type *LamC* transgene across 30 vials with 0.05% DMSO. Of these, 861 developed into viable adults (95.45% viability), which is consistent with the adult viability observed for this genotype in the absence of DMSO [20,54,56]. To confirm that DMSO did not have a therapeutic effect, 1133 total progeny expressing *LamC* R264Q across 35 independent vials were treated with 0.05% DMSO. These progeny died at the pupal stage as expected, with zero adult survivors. This is consistent with our previous data in the absence of drug, where *LamC* R264Q expression resulted in completely penetrant pupal lethality [20]. These experiments confirm that DMSO is neither toxic nor therapeutically beneficial in this context, supporting the conclusion that the emergence of adult flies following drug treatment indicates a positive effect of the drug and not the vehicle used in the screen.

Using this approach, we successfully screened 1433 drugs (87 vials had unsuccessful matings) from the Prestwick Chemical Library for rescue of lethality caused by muscle-specific expression of *LamC* R264Q (Supplemental Table S1). Treatment with 68 drugs resulted in at least one viable adult for a hit rate of 4.75% (Figure 2B). The number of viable adults per vial containing each hit drug ranged from one to eight, with one to three being most common. The number of dead pupae per vial averaged 32. A list of the 68 positive hit drugs, their molecular target or the biological process they target according to the compound key provided by Prestwick Chemical, and their action against that target is provided in Table 1. Note that many of the drugs with a defined molecular target have an orthologue in Drosophila that is expressed at the larval and/or pupal stage (Table 1) (FlyBase, FB2026_02, released 18 June 2026).

### 2.2. Structural Clustering of Positive Hit Compounds Identified Dihydropyridine L-Type Voltage-Gated Calcium Channel Antagonists Among the Drugs That Rescued Lethality

To prioritize the 68 hit drugs for follow-up testing, we hypothesized that drugs with similar structures likely have the same or similar molecular targets, making assessment of structural similarity a way to identify enriched biological targets of the drugs in our hit compound list. We compared the structures of the hit drugs using the open-source cheminformatics tool RDKit (https://www.rdkit.org (accessed on 15 September 2025)) [76,77,78]. RDKit can be used to generate molecular fingerprints, or binary code representations of a compound, which can be compared to each other using similarity indices such as the Tanimoto similarity index to generate numerical measures of the similarity between every pairwise comparison of compounds in a data set [78,79,80]. The Tanimoto similarity index ranges from 0 to 1, where a value of 0 indicates completely different molecules and a value of 1 indicates identical molecules [78]. All similarity indices for a given data set can then be clustered using algorithms such as the Butina clustering algorithm to identify larger clusters of compounds that all reach a certain threshold of similarity to each other while being different from all other molecules in the data set [81].

**Figure 3:**
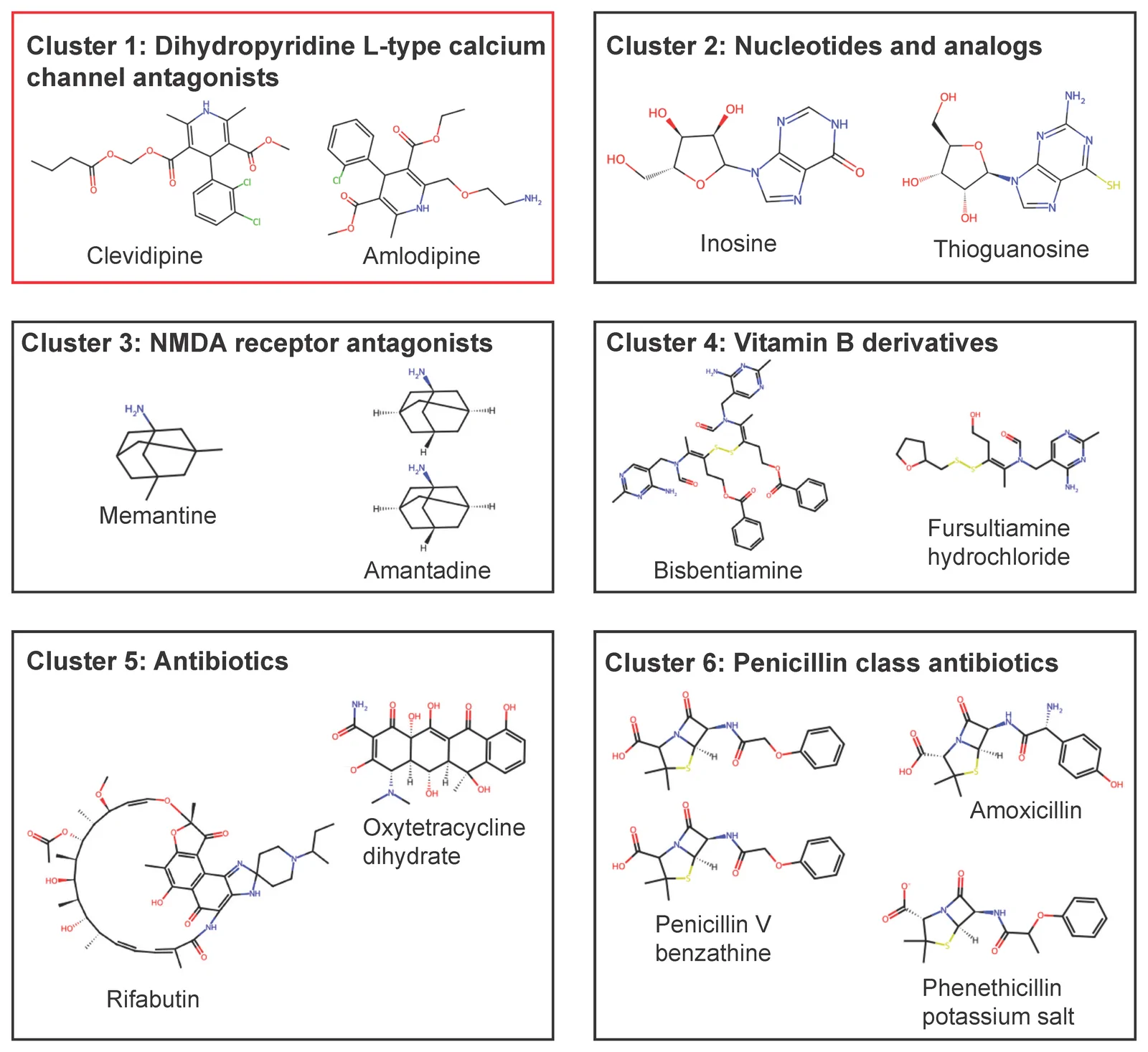
Molecular fingerprinting revealed six clusters of structurally similar compounds among the positive hit drugs. Six clusters of compounds with at least 75% structural similarity to each other were identified from the primary screen hit drug list through comparisons of molecular fingerprints generated in RDKit using Tanimoto similarity and Butina clustering.

When this workflow was applied to our 68 hit compounds, it identified six clusters of at least two compounds that had a Tanimoto similarity of 0.75 or more, meaning all compounds in each cluster are at least 75% structurally similar to each other (Figure 3). In total, 13 compounds were included in the clusters, with five clusters containing two compounds and one cluster containing three. Compounds in each cluster were indeed functionally related, with the six clusters including two classes of antibiotics, nucleotides, NMDA receptor antagonists, vitamin B derivatives, and dihydropyridine (DHP) L-type calcium channel (LTCC) antagonists (Figure 3).

Of the six clusters, the one with the clearest connection to muscle function comprised the DHP LTCC antagonists. LTCC antagonists are readily available drugs that are used to treat various cardiac conditions, most notably hypertension [82,83]. These drugs are structurally classified into DHPs and non-DHPs, which block LTCCs through different binding sites, variable affinities, and with slightly different physiological effects [83,84]. The DHPs include clevidipine and amlodipine identified in our screen (Table 1), as well as many others [84]. The two most notable non-DHPs are verapamil, which is classified as a phenylalkylamine, and diltiazem, which is a benzothiazepine [84]. Importantly, our screen also identified ethaverine (Table 1), which is a non-DHP LTCC antagonist that is structurally similar to verapamil [85]. This reveals a limitation of structural clustering as a means of hit prioritization, as not all compounds with the same molecular target have similar structures.

### 2.3. Secondary Testing Verified DHP Calcium Channel Blockers as Positive Hits in the LamC R264Q Model

Given the physiological differences in the actions of DHP and non-DHP calcium channel blockers [84,86] and the fact that we identified two structurally similar DHPs compared to one non-DHP in our screen (Table 1), we prioritized the DHPs for secondary testing. To confirm clevidipine and amlodipine as hits in our screen, we purchased both drugs from a new source outside of Prestwick Chemical to control for batch effects and impurities or altered drug conformations that potentially could be present in the drug solution used in our screen (Table S2). Like in the primary screen, these newly purchased drug aliquots were dissolved at 10 mM in DMSO and diluted to 1 mM and 10% DMSO in PBS. Newly purchased clevidipine and amlodipine were again fed to larvae expressing *LamC* R264Q in the larval body wall muscle through a method largely identical to the primary screen at concentrations ranging from 0.5 to 50.0 µM (0.5, 1.25, 2.5, 3.75, 5.0, 12.5, 25.0, 37.5, 50.0 µM). In total, 58 vials were treated with clevidipine, and eight vials produced at least one live adult at concentrations ranging from 0.5 to 37.5 µM (0.5, 1.25, 2.5, 5.0, 12.5, 25.0, 37.5 µM). A total of 67 vials were treated with amlodipine, and six produced at least one adult at concentrations ranging from 2.5 to 37.5 µM (2.5, 3.75, 5.0, 10.0, 37.5 µM). In parallel, 52 additional vials were treated with vehicle 0.05% DMSO as a control, and none yielded any live adults, consistent with the controls in the primary screen (Figure S1). These results confirm that DHP L-type calcium channel blockers partially rescue pupal lethality caused by *LamC* R264Q expression.

### 2.4. DHP Calcium Channel Blockers Selectively Rescue Lethality Caused by LamC R264Q, R264W, and R237P

It is well established that different lamin amino acid substitutions result in different molecular defects despite converging on a similar muscular dystrophy phenotype [52,54,58]. Given this, a treatment that rescues muscle defects caused by one lamin substitution may not be universally effective against muscle defects caused by other lamin substitutions. To determine the broad applicability of the rescue of lethality we observed in the *LamC* R264Q model following DHP treatment, we tested the DHPs in six additional Drosophila models expressing different mutant lamins (Figure 1). These include the LamC S72P, R237P, R264W, H545P, M553R, and R564P substitutions, which are spread across the full length of the lamin protein and are all orthologous to pathogenic mutations in humans [20,52,54,56,87,88]. The Drosophila models expressing *LamC* R237P, R264W, M553R, and R564P transgenes have been previously characterized, and independent expression of these mutant lamins in larval body wall muscles results in completely penetrant pupal lethality akin to what is caused by *LamC* R264Q [20,52,54,56]. To characterize the *LamC* S72P and H545P models, we expressed the mutant lamin transgenes specifically in the larval body wall muscles using the *C57*-Gal4 driver [74,75]. Expression of these mutant *LamC* transgenes also resulted in complete death at the pupal stage, with expression of the previously characterized *LamC* M553R used as a positive control (Table S3) [52]. Given that expression of these six mutant lamins results in complete loss of adult viability, as did the expression of *LamC* R264Q, the calcium channel blockers were tested in these models.

**Figure 4:**
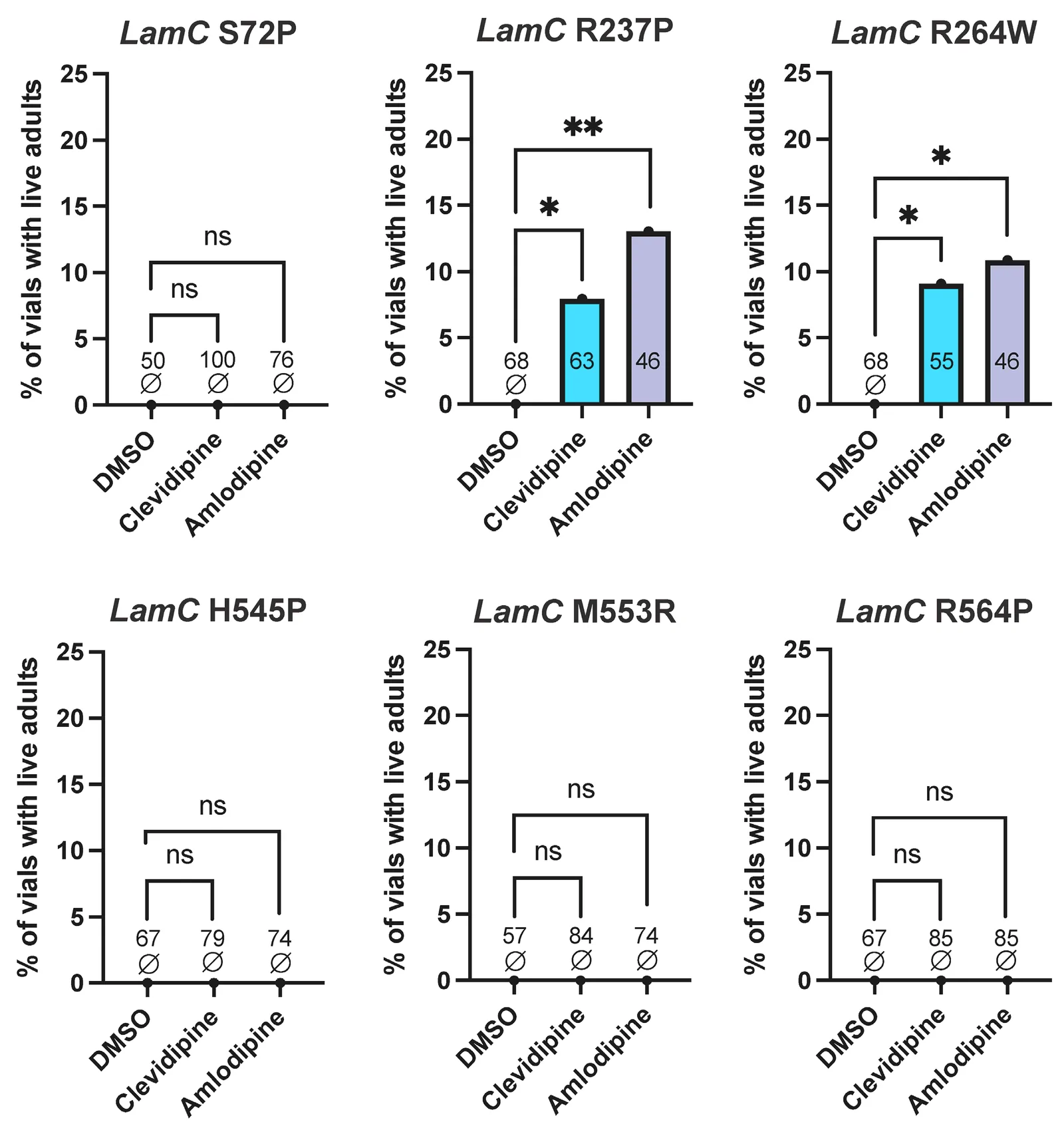
Mutant *LamC*-specific rescue of lethality by clevidipine and amlodipine. Larvae with muscle-specific expression of different mutant lamins (indicated at the top of each graph) were tested for rescue of lethality upon treatment with either clevidipine or amlodipine. The total number of vials scored per genotype is indicated. The percentage of live adults was plotted, and statistical significance was determined using Fisher’s Exact Test (GraphPad Prism). *, *p* < 0.05; **, *p* < 0.01.

Drosophila larvae expressing these additional mutant lamins in the larval body wall muscles were independently treated with clevidipine, amlodipine, or DMSO only as a control in an identical fashion to the secondary screen. Clevidipine and amlodipine treatment resulted in vials producing live adults only following expression of either the *LamC* R237P or R264W transgene. Of the 63 vials of larvae expressing *LamC* R237P treated with clevidipine, five vials produced live adults at drug concentrations ranging from 1.25 µM to 5 µM (1.25, 2.5, 3.75, 5.0 µM). Similarly, 46 vials of larvae expressing *LamC* R237P were treated with amlodipine, and six vials yielded live adults at concentrations ranging from 1.25 µM to 25 µM (1.25, 3.75, 12.5, 25.0 µM). Of 55 vials of larvae expressing *LamC* R264W treated with clevidipine, five produced live adults at concentrations ranging from 0.5 to 5 µM (0.5, 1.25, 2.5, 3.75, 5.0 µM). In addition, 46 vials of larvae expressing *LamC* R264W were treated with amlodipine, and five produced live adults at concentrations ranging from 2.5 to 37.5 µM (2.5, 3.75, 5.0, 25.0, 37.5 µM). Similar numbers of vials of larvae expressing the S72P, H545P, M553R, and R564P mutant lamins were treated with each drug, and none produced live adults (Figure 4). Thus, the ability of DHP L-type calcium channel blockers to restore adult viability following mutant lamin expression extends beyond the R264Q mutant lamin used in our initial screen to include *LamC* R264W and R237P. However, the rescue is not universal, as the drugs provided no apparent benefit to larvae expressing *LamC* S72P, H545P, M553R, and R564P, suggesting a need for personalized treatments for individuals with *LMNA*-MD [52,54,58].

### 2.5. Non-DHP Calcium Channel Blockers Rescue Lethality Caused by LamC R264Q, R264W, and R237P

LTCC blockers are broadly divided into DHPs, such as clevidipine and amlodipine, and non-DHPs [83,84,86]. Given that our primary screen identified both types as positive hit drugs, we next sought to determine whether our previous findings on the DHP calcium channel blockers extended to non-DHPs [85]. While both drug classes inhibit L-type calcium channels, they have different physiological effects, binding affinities with various LTCCs, and primary binding sites in humans [83], necessitating follow-up testing. While the non-DHP ethaverine was identified in our primary screen, it is structurally similar to verapamil [85]. Verapamil and diltiazem are the most widely prescribed non-DHPs and were thus prioritized for testing [84,86].

**Figure 5:**
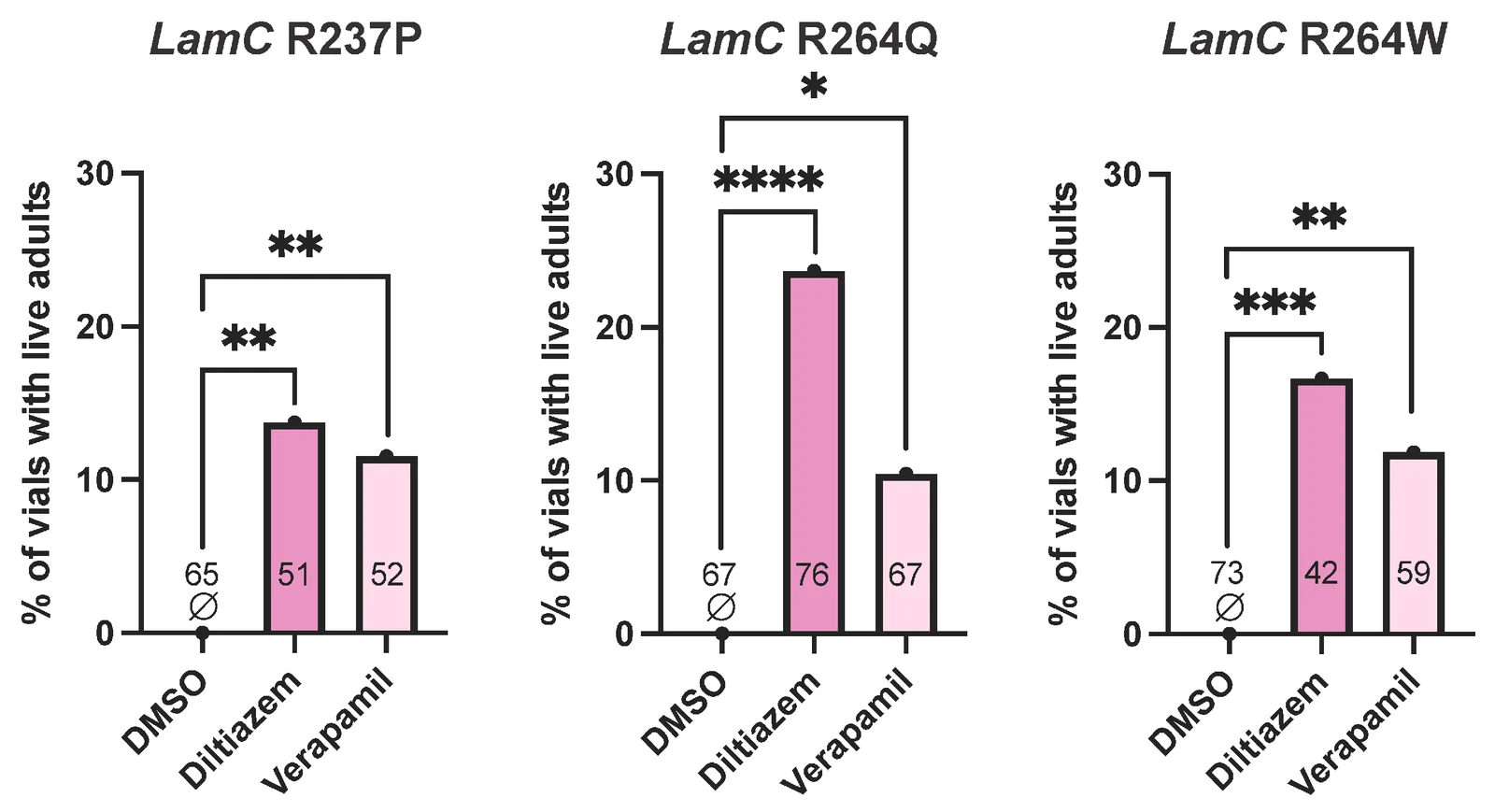
Non-DHP calcium channel inhibitors rescue lethality caused by mutant lamins. Larvae with muscle-specific expression of different mutant lamins (indicated at the top of each graph) were tested for rescue by treatment with either diltiazem or verapamil. The total number of vials scored per genotype is indicated. The percentage of live adults was plotted, and statistical significance was determined using Fisher’s Exact Test (GraphPad Prism). *, *p* < 0.05; **, *p* < 0.01; ***, *p* < 0.001; ****, *p* < 0.0001.

Given that the DHP calcium channel blockers rescued lethality caused by expression of the *LamC* R237P, R264Q, and R264W transgenes, verapamil and diltiazem were tested in these three models to determine whether non-DHPs could also restore adult viability. All three models were treated with verapamil and diltiazem at concentrations ranging from 0.5 to 50.0 µM (0.5, 1.25, 2.5, 3.75, 5.0, 12.5, 25.0, 37.5, 50.0 µM) through the same methods as DHP treatment. In total, 51 vials containing larvae expressing *LamC* R237P, 76 vials containing larvae expressing *LamC* R264Q, and 42 vials containing larvae expressing *LamC* R264W were treated with diltiazem, while 52, 67, and 59 vials of larvae expressing each mutant lamin, respectively, were treated with verapamil. Treatment with diltiazem and verapamil was effective in all three models (Figure 5). Diltiazem treatment resulted in seven vials yielding adults following expression of *LamC* R237P, 18 vials yielding adults following expression of *LamC* R264Q, and seven vials yielding adults following expression of *LamC* R264W. Verapamil treatment resulted in six vials yielding adults following expression of *LamC* R237P, seven vials yielding adults following expression of *LamC* R264Q, and seven vials yielding adults following expression of *LamC* R264W (Figure 5). The efficacy of diltiazem and verapamil in the same Drosophila models in which the DHPs were effective suggests that L-type calcium channel inhibition rescues lethality caused by mutant lamins regardless of the mechanism by which the channel is targeted. These findings bolster the case for the L-type calcium channel as a potential therapeutic target in *LMNA*-MD.

### 2.6. Calcium Channel Blocker Treatment Reduces Intracellular Calcium in Muscles of Larvae Expressing LamC R264Q

Given that we obtained rescue of lethality upon treatment with the calcium channel blockers, we wanted to determine if the drug treatment improved muscle physiology. Of the flies expressing mutant lamins that responded to clevidipine, those expressing *LamC* R237P showed thin and fragile muscles compared to controls upon dissection (Figure S2). Therefore, we performed a morphometric analysis on larval body wall muscle from larvae expressing wild-type *LamC* and *LamC* R237P with and without clevidipine treatment. Flies expressing *LamC* R564P were used as a positive control since our prior studies demonstrated that this mutant lamin caused decreased muscle size [56]. Larval body wall muscles were fixed and stained with phalloidin (Figure S2). Larval body wall muscles have a characteristic arrangement of muscles, allowing the same muscle to be identified and measured across larvae [89,90]. Quantification of the muscle width showed that larvae expressing *LamC* R564P had reduced muscle width relative to the control, consistent with our prior findings [56]. The muscles of larvae expressing *LamC* R237P showed reduced muscle width relative to the control, and this did not change upon treatment with clevidipine (Figure S2). Thus, the benefits of clevidipine are not through enhancing muscle physiology.

**Figure 6:**
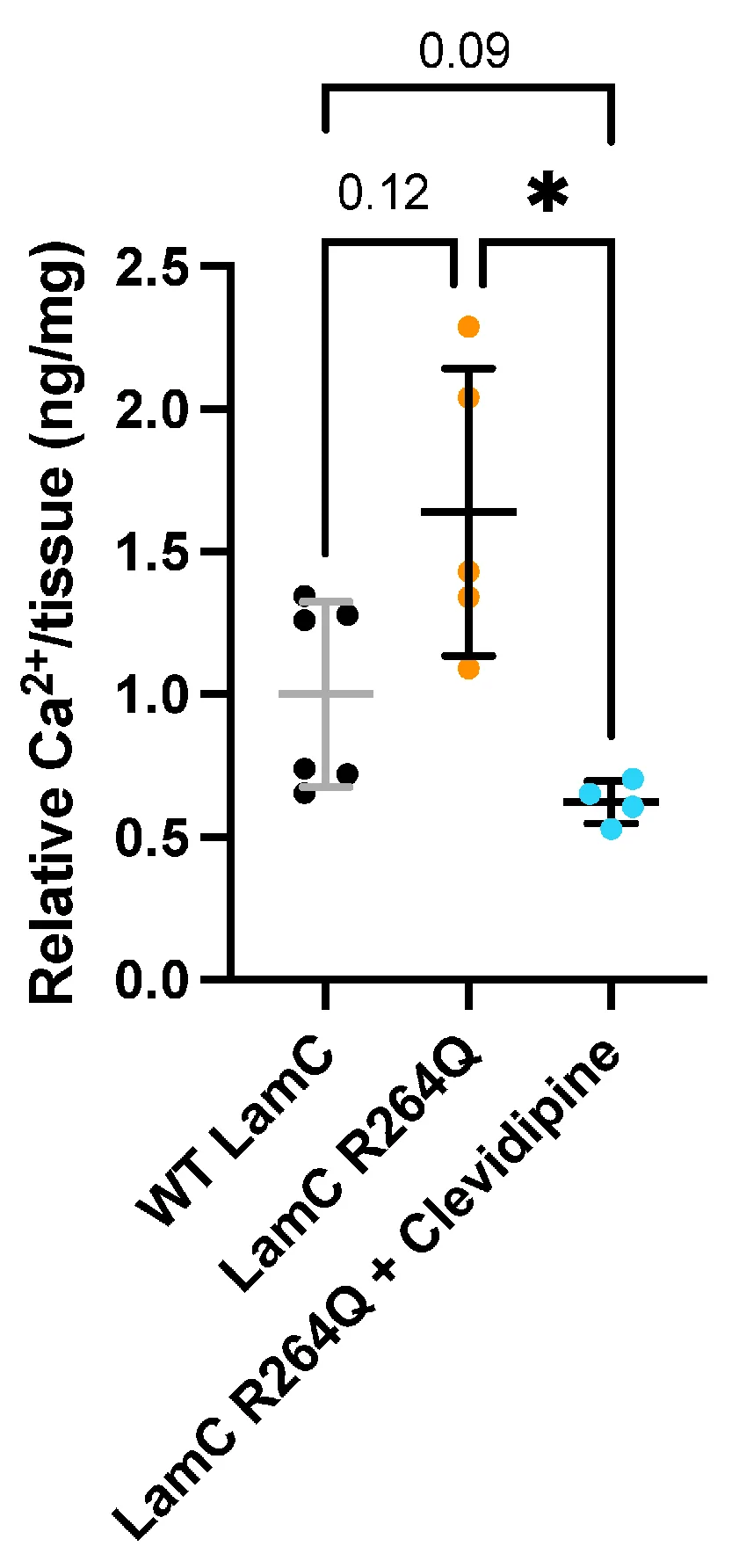
Clevidipine reduces levels of calcium in larval muscles expressing *LamC* R264Q. Four to six independent groups of 15 larvae per genotype were used for calcium assays. Muscles were dissected, treated with detergent, homogenized, centrifuged, and the supernatant collected for calcium measurements using a colorimetric assay (Abcam, Cambridge, UK). Calcium was elevated in larvae expressing *LamC* R264Q relative to larvae expressing wild-type *LamC*, with values trending towards significance. Clevidipine-treated larvae expressing *LamC* R264Q showed a reduction in intracellular calcium compared to those untreated. Error bars indicate standard deviation, and the *p* values were calculated by a one-way ANOVA with Dunnett’s correction (GraphPad Prism). *, *p* < 0.05.

Since we obtained rescue of viability upon treatment with calcium channel blockers, we aimed to determine their mechanism of action in Drosophila muscle. We dissected muscles from larvae expressing wild-type *LamC* and *LamC* R264Q with and without treatment with 1.0 and 5.0 μM clevidipine and measured intracellular calcium. Muscle fillets were prepared from wandering third instar Drosophila larvae, treated with detergent, homogenized, centrifuged, and the supernatant used in a colorimetric assay (Abcam, Cambridge, UK). The results showed that larvae expressing *LamC* R264Q had increased calcium compared to the control, albeit variable and trending towards significance (Figure 6). Extracts from clevidipine-treated larvae expressing *LamC* R264Q showed lower calcium than those from untreated larvae expressing *LamC* R264Q, trending below the levels in larvae expressing wild-type *LamC* (Figure 6). Thus, *LamC* R264Q appears to generally increase calcium levels in muscle and clevidipine reduces those levels, consistent with clevidipine functioning on the Drosophila calcium channel Ca-α1D expressed in muscle [91,92].

## 3. Discussion

We completed an unbiased drug screen to identify compounds that rescue lethality caused by muscle-specific expression of mutant lamins in Drosophila. Of the 1433 drugs screened, 68 rescued adult viability. Prioritization of these hit compounds by structural similarity clustering revealed six groups of two to three compounds that were at least 75% structurally similar, suggesting engagement of the same biological target. We selected the dihydropyridine (DHP) L-type calcium channel antagonists clevidipine and amlodipine for further analysis given the role of calcium in muscle function [93,94,95]. Calcium is the key driver of muscle excitation–contraction coupling (ECC), and increased intracellular calcium is a common mechanism known to underlie many types of muscular dystrophy [82,83]. Elevated calcium influx can lead to abnormal ECC, calcium-dependent protease (calpain) activation, mitochondrial dysfunction, and increased production of reactive oxygen species, all of which are factors that can lead to muscle dysfunction and, ultimately, myofiber necrosis [82,96,97].

Support for this calcium hypothesis as a common pathogenic mechanism of muscular dystrophy has been derived from manipulations of calcium flux in several animal models and analysis of patient samples. There is ample evidence that myofibers in muscular dystrophy patients and several mouse models of muscular dystrophy have elevated levels of intracellular cytoplasmic calcium [82,96]. Reducing calcium release from the sarcoplasmic reticulum (SR) or increasing calcium reuptake into the SR rescues muscle phenotypes and improves dystrophic pathology in *Sgcd*-null mice, a model of LGMD, and *mdx* mice, a model of DMD [98,99,100,101]. Direct calcium influx across the sarcolemma independent of the SR involves transient receptor potential channels (TRPCs), voltage-independent channels that open to allow calcium influx in response to stretch or decreased SR calcium content [82,102]. An increased amount of calcium enters the cytoplasm of muscle cells through these channels in dystrophic versus non-dystrophic muscle [102]. Overexpression of dominant-negative TRPCs that are not permeable to calcium rescues dystrophic pathology in mouse models of muscular dystrophy, while overexpression of wild-type TRPCs induces dystrophic pathology in wild-type mice [103]. These studies robustly demonstrate that increased intracellular calcium contributes to the pathogenesis of muscular dystrophy, and correcting dysregulation of calcium homeostasis has therapeutic potential [82].

An attractive therapeutic target for addressing this dysregulated calcium homeostasis is LTCCs, as they are readily targetable by DHP and non-DHP calcium channel blockers [104]. In mammals, the LTCCs are Ca_v_1.1, 1.2, 1.3, and 1.4 encoded by *CACNA1S*, *C*, *D*, and *F*, respectively [105,106]. Ca_v_1.1 is the skeletal muscle-specific LTCC [93], Ca_v_1.2 is the primary LTCC in cardiomyocytes and vascular smooth muscle [104], Ca_v_1.3 is the primary LTCC in cells of the sinoatrial and atrioventricular nodes of the heart [104], and Ca_v_1.4 is largely restricted to the retina and plays no role in striated muscle [104]. Biochemically, DHPs have a higher affinity for inactivated LTCCs and bind the external, lipid-facing surface of the channel pore to stabilize the inactive channel state [104,107,108]. Conversely, the non-DHPs verapamil and diltiazem have higher affinity for the open channel state and bind the central cavity of the channel pore on the intracellular side, directly blocking the pore [104,107,108]. Physiologically, DHPs primarily target Ca_v_1.2 in vascular smooth muscle and cause vasodilation, lowering blood pressure [84,104,109]. Ca_v_1.3 is five- to ten-fold less sensitive to DHPs than Ca_v_1.2, while the non-DHPs target Ca_v_1.2 and Ca_v_1.3 equivalently [84,104,109]. Inhibition of Ca_v_1.3 in the sinoatrial and atrioventricular nodes of the heart by the non-DHPs reduces cardiac conduction and contractility, making them useful for treating cardiac arrhythmias in addition to hypertension [84,104,109].

Despite these primary actions on Ca_v_1.2 and Ca_v_1.3 in vascular smooth muscle and the heart, both DHPs and non-DHPs have been extensively tested for therapeutic benefit in animal models of muscular dystrophy based on their ability to target Ca_v_1.1 with nanomolar affinity and the ready availability of the drugs [104,110,111]. However, the LTCC appears to be a poorer target in skeletal muscle, as the skeletal muscle LTCC is mechanically coupled to RyR1 and external calcium does not need to pass through the LTCC to induce RyR1 opening [82]. Despite this, the non-DHP verapamil and diltiazem and the DHP nifedipine improved dystrophic phenotypes, diaphragm muscle pathology, and muscle strength in *mdx* mice while decreasing intracellular calcium content [112,113,114]. In *Dysf*-null mice, a model of LGMD, increased calcium leak through RyR1 is a known mechanism of pathogenesis [115]. DHPs, specifically nifedipine, and the non-DHP verapamil can reduce this leak by modifying the interaction between Ca_v_1.1 and RyR1 [116,117,118]. In myotonic dystrophy, increased skipping of *CACNA1S* exon 29 results in a Ca_v_1.1 isoform highly deleterious to muscle function, altering the gating of RyR1 and leading to significantly increased calcium influx [119,120]. The reduced function of muscles expressing this Ca_v_1.1 isoform is attenuated by the non-DHP verapamil [120].

While mammals have four LTCCs, Ca-α1D is the single LTCC in Drosophila [121]. Mutations in the *Ca-α1D* gene cause several phenotypes indicative of muscle contraction defects in embryos, larvae, and adult flies [91]. Calcium currents through this channel in larval muscle are sensitive to DHPs, diltiazem, and verapamil [92]. Ca-α1D is also the primary calcium channel expressed in the fly myocardium, and knockdown of *Ca-α1D* specifically in the fly heart abolishes cardiac contraction [122]. Calcium currents through Ca-α1D in the Drosophila heart are also DHP-sensitive [122]. Thus, Drosophila Ca-α1D shares physiological similarities with mammalian Ca_v_1.1, 1.2, and 1.3 and has conserved roles in cardiac and muscle function [123]. Based on our findings of intracellular calcium in muscle in these models (Figure 6), we infer that clevidipine is likely functioning through the Drosophila Ca-α1D channel; however, this requires further investigation.

Consistent with the conservation of the LTCC in Drosophila and the success of LTCC blockers in rescuing muscle phenotypes in other animal models of muscular dystrophy, follow-up testing of clevidipine and amlodipine from an alternative source confirmed the ability of these DHPs to restore adult viability following expression of the R264Q mutant lamin in our studies. Clevidipine and amlodipine treatment rescued lethality caused by three mutant lamins but not four others tested, demonstrating that the positive effect of these compounds is not confined to a single amino acid substitution. However, the positive effect is also not universal across the mutational spectrum of laminopathies. The fact that the non-DHPs verapamil and diltiazem were also effective at restoring viability in the three mutants rescued by the DHPs demonstrates that the therapeutic benefit of blocking the L-type calcium channels is not confined to an individual drug class. Collectively, these results support L-type calcium channel antagonists as potential drug repurposing candidates in at least some cases of *LMNA*-MD.

While our study and those of others support the efficacy of LTCC blockers in animal models of muscular dystrophy, clinical trials assessing their benefits for individuals with DMD showed mixed results [82,112]. Verapamil is effective in improving muscle strength but results in significant cardiac side effects [124]. Diltiazem has been assessed in multiple trials with opposing results [125,126]. Nifedipine, a DHP, was deemed to have no significant benefit in DMD [127]. The lack of a clear effect in patients as opposed to animal models could be due to several factors, including differences in drug metabolism, variations in dosing, and heterogeneity in clinical trial cohorts [112]. While further studies are necessary to clarify the mechanisms underlying the variability in outcomes in humans, the success of these compounds in animals supports the view that pathological calcium influx is a targetable mechanism in muscular dystrophy.

Muscular dystrophies in which altered calcium homeostasis has been implicated as a mechanism of pathogenesis are primarily caused by mutations in genes encoding cytoskeletal proteins or proteins that connect the cytoskeleton and extracellular matrix and contribute to intracellular force transmission [82,96,128,129]. All the genes encoding nuclear envelope proteins that are implicated in muscular dystrophy are either part of the Linker of Nucleoskeleton and Cytoskeleton (LINC) complex or directly interact with LINC complex components [128,130]. This raises the possibility that muscular dystrophy-associated alterations to nuclear envelope proteins could dysregulate calcium homeostasis through a loss of muscle membrane integrity driven by cytoskeletal defects akin to those caused by mutations in genes encoding cytoskeletal components directly [128]. It is known that pathogenic mutations in lamins can cause mislocalization of other nuclear envelope proteins, including the LINC complex, leading to impaired nucleo-cytoskeletal coupling, mislocalization of cytoskeletal proteins in the perinuclear region, and impaired intracellular force transmission [1,20,54,58,131,132]. While further studies are needed to define the connection between calcium regulation and lamins, our drug screen has provided new insights on disease mechanisms and potential treatments for *LMNA*-MD.

## 4. Materials and Methods

### 4.1. Drosophila Culture

Drosophila stocks were maintained on a sucrose and cornmeal-based medium at 25 °C unless otherwise stated. The generation of *LamC* transgenic stocks was previously described, except for *LamC* H545P and S72P [20,52,54,56]. Muscle-specific expression of the mutant *LamC* transgenes was achieved by crossing the transgenic lines to the *C57*-Gal4 driver, which expresses the yeast Gal4 specifically in larval body wall muscles [74,75]. The resulting progeny express the *LamC* transgene exclusively in the larval body wall muscles.

### 4.2. Primary Drug Screen

The Prestwick Chemical Library (version 20, Illkirch, France) was purchased commercially. The library contained 1520 compounds dissolved at 10 mM in DMSO. Drugs were diluted to 1 mM in 10% DMSO with phosphate-buffered saline in 96-well plates (USA Scientific, cat# 5665-1101, Ocala, FL, USA). In total, 10 µL of each drug was added to a 15 mL vial (Sigma Aldrich, cat# Z617865-1600EA, St. Louis, MO, USA) containing 2 mL liquid Drosophila culture medium, generating a final drug concentration of 5 µM and 0.05% DMSO. Next, 10 µL of 10% DMSO was added to separate vials with 2 mL of Drosophila medium as a control. The food was allowed to solidify at 17 °C overnight. Compounds were screened in batches of 40. Two *C57*-Gal4 female virgins and two *LamC* R264Q transgenic males were placed in each vial, allowed to mate, and removed after four days. Offspring expressing *LamC* R264Q in larval body wall muscles consumed the drug-containing food throughout development at 25 °C, pupated, and adult viability was assessed 14 days post mating. Drugs were deemed “hits” if they resulted in any restoration of adult viability, given that expression of *LamC* R264Q is well established to result in completely penetrant pupal lethality [20]. The number of live adults per hit vial ranged from one to eight, and the number of dead pupae remaining per vial averaged approximately 30.

### 4.3. Molecular Fingerprinting and Structural Clustering

The molecular similarity of the hit drugs was computed using the RDKit package followed by Butina clustering according to standard methods [76,78,81]. RDKit is an open-source cheminformatics tool (https://www.rdkit.org (accessed on 15 September 2025), https://doi.org/10.5281/zenodo.6961488) that was run using Python 3.1.3. SMILES strings for all screened molecules were provided as part of the purchase of the Prestwick Chemical Library. SMILES strings of the 68 hit drugs identified in the primary screen were imported into RDKit for processing. Molecular fingerprints were generated as bit vectors and, subsequently, a bulk Tanimoto similarity calculation was employed to determine the molecular similarity for all pairwise comparisons of compounds. The dissimilarity (distance) matrix was then calculated as 1 minus the Tanimoto similarity for each pairwise comparison. The dissimilarity coefficients for each pairwise comparison were further assessed using the Butina clustering approach, using a cutoff threshold of 0.25, to generate clusters of multiple structurally similar drugs.

### 4.4. Secondary Screen and the Assessment of Broad Efficacy

A list of compounds used for the secondary screen and the assessment of broad efficacy is provided in Table S1. Compounds were dissolved at 10 mM in DMSO and diluted to 1 mM in 10% DMSO in phosphate-buffered saline immediately prior to use. Compounds were added to 15 mL vials (Sigma Aldrich, cat# Z617865-1600EA, St. Louis, MO, USA) at appropriate volumes to generate final concentrations between 0.5 and 50.0 µM (0.5, 1.25, 2.5, 3.75, 5.0, 12.5, 25.0, 37.5, 50.0 µM). 10 µL of 10% DMSO in phosphate-buffered saline alone was added to additional vials as a vehicle-only control. Adult viability 14 days post mating was assessed identically to the primary screen. The number of live adults per vial ranged from one to five. The number of vials producing or not producing live adults was compared between drug-treated flies and those treated with DMSO alone as a control in a contingency table. Statistical significance was assessed using Fisher’s Exact Test in GraphPad Prism (v10.6.1, GraphPad Software, San Diego, CA, USA).

### 4.5. Quantification of Adult Viability

Flies possessing wild-type *LamC* and either *LamC* H545P or S72P transgenes were crossed with flies possessing the muscle-specific *C57*-Gal4 driver for quantitative assessment of adult viability. After four days, the adults were removed from the vials, and progeny were allowed to develop. The resulting dead pupae and live adults were counted after 14 days. The percent adult viability was calculated as [# living adults/(# living adults + # dead pupae)] × 100. The total number of progeny ranged from 300 to 695 per genotype.

### 4.6. Measurements of Intramuscular Calcium

Muscle tissue was dissected from 15 third instar larvae per sample, with four to six independent samples analyzed per genotype. The muscles were weighed, washed, treated with detergent, homogenized, centrifuged, and the supernatant mixed with a chromogenic reagent that detects calcium (#ab102505, Abcam, Cambridge, UK). Optical density was detected using a 96-well plate reader (Synergy NEO, Agilent BioTek, Santa Clara, CA, USA) with the absorbance read at 575 nm. A dilution of calcium standards at known concentrations provided by the manufacturer was used to generate a standard curve, allowing for calcium concentrations/mg tissue to be calculated. For each independent trial, values were normalized to those of larvae expressing wild-type *LamC*, set to 1, so that independent trials could be compared (Figure 6). The average and standard deviations were plotted using GraphPad Prism (v10.6.1, GraphPad Software, San Diego, CA, USA) and a one-way ANOVA with Dunnett’s correction was used to determine statistical significance.

### 4.7. Morphometric Analysis of Muscle Fibers

Larval body wall muscles were dissected, fixed with formaldehyde, and stained with phalloidin as previously described [20,52,56]. Muscles were imaged using a Leica Thunder microscope (Leica, Wetzlar, Germany). Muscles six and seven were identified in each larval muscle fillet and width measurements were made using the straight-line selection tool in Fiji software (https://imagej.net/software/fiji/ (accessed on 16 August 2026), ImageJ2, v2.16.0/1.54p) [133]. Muscles from three to six larvae were measured per genotype. Averages with standard deviation were plotted, and a one-way ANOVA was performed with Dunnett’s correction (GraphPad Prism v10.6.1, GraphPad Software, San Diego, CA, USA).

## Figures and Tables

**Table 1:** The primary screen identified 68 positive hits.

Chemical Name	CAS Number	Human Therapeutic Effect	Molecular Target or Process	Target Mechanism	Ortholog of Target Expressed in Larvae/Pupae
**Xylazine**	7361-61-7	Analgesic	Adrenergic Alpha 2 receptor	agonist	Yes
**Ufenamate**	67330-25-0	Analgesic	Cyclooxygenase-1, 2	inhibitor	Yes
**Mefenamic acid**	61-68-7	Analgesic	Cyclooxygenase-2	inhibitor	Yes
**Niridazole**	61-57-4	Anthelmintic	DNA biosynthetic process	inhibitor	-
**Hycanthone**	3105-97-3	Anthelmintic	DNA-(apurinic or apyrimidinic site) lyase	inhibitor	Yes
**Memantine hydrochloride**	41100-52-1	Anti-Alzheimer	Glutamate receptor	antagonist	Yes
**Inosine**	58-63-9	Anti-Alzheimer	reactive oxygen species biosynthetic process	inhibitor	Yes
**Fursultiamine hydrochloride**	2105-43-3	Anti-Alzheimer	Vitamin B1	N/A	No
**Nalmefene hydrochloride**	58895-64-0	Anti-opioid	Opioid receptors mu/kappa/delta	antagonist	Yes
**N-Acetylaspartylglutamic acid**	3106-85-2	Antiallergic	Metabotropic glutamate receptor 3	agonist	Yes
**Bisbentiamine**	2667-89-2	Antianemic	Not known	N/A	-
**Acebutolol hydrochloride**	34381-68-5	Antianginal	Adrenergic beta-1 receptor	antagonist	Yes
**Benzbromarone**	3562-84-3	Antianginal	Uric acid uptake	inhibitor	Yes
**Terbutaline hemisulfate**	23031-32-5	Antiasthmatic	Adrenergic beta-2 receptor	agonist	Yes
**Cromolyn disodium salt**	15826-37-6	Antiasthmatic	G-protein coupled receptor 35	agonist	No
**Repirinast**	73080-51-0	Antiasthmatic	Histamine release	inhibitor	-
**Seratrodast**	112665-43-7	Antiasthmatic	Thromboxane A2 receptor	antagonist	No
**Oxytetracycline dihydrate**	6153-64-6	Antibacterial	30S unit	inhibitor	No
**Sulfaquinoxaline sodium salt**	967-80-6	Antibacterial	Bacterial dihydrofolate synthetase	inhibitor	No
**Amoxicillin**	26787-78-0	Antibacterial	Bacterial transpeptidase	inhibitor	No
**Dicloxacillin sodium salt hydrate**	13412-64-1	Antibacterial	Bacterial transpeptidase	inhibitor	No
**Cefuroxime sodium salt**	56238-63-2	Antibacterial	Bacterial transpeptidase	inhibitor	No
**Phenethicillin potassium salt**	132-93-4	Antibacterial	Bacterial transpeptidase	inhibitor	No
**Rifabutin**	72559-06-9	Antibacterial	DNA-directed RNA polymerase subunit beta	inhibitor	Yes
**Colistin sulfate**	1264-72-8	Antibacterial	Not known	-	-
**Penicillin V benzathine**	5928-84-7	Antibacterial	Peptidoglycan synthesis	inhibitor	No
**Oxolinic acid**	14698-29-4	Antibacterial	Topoisomerase II	inhibitor	Yes
**Ticlopidine hydrochloride**	53885-35-1	Anticoagulant	Purinergic P2Y12 receptor	antagonist	Yes
**Levetiracetam**	102767-28-2	Anticonvulsant	Synaptic vesicle glycoprotein 2A	agonist	Yes
**Isocarboxazid**	59-63-2	Antidepressant	Monoamine oxidase	inhibitor	Yes
**Venlafaxine**	93413-69-5	Antidepressant	Serotonin transporter, NE transporter	inhibitor	Yes
**Milnacipran hydrochloride**	101152-94-7	Antidepressant	Serotonin transporter, NE transporter	inhibitor	Yes
**Dosulepin hydrochloride**	897-15-4	Antidepressant	Tumor protein p53	agonist	Yes
**Palonosetron hydrochloride**	135729-62-3	Antiemetic	Serotoninergic 5-HT3 receptor	antagonist	Yes
**Butoconazole nitrate**	64872-77-1	Antifungal	Cytochrome P450 51	inhibitor	Yes
**Posaconazole**	171228-49-2	Antifungal	Cytochrome P450 51	inhibitor	Yes
**Butylparaben**	94-26-8	Antifungal	Not known	-	-
**Tolnaftate**	2398-96-1	Antifungal	Squalene monooxygenase	inhibitor	No
**Chlormidazole**	3689-76-7	Antifungal	synthesis of ergosterol	inhibitor	-
**Pheniramine maleate**	132-20-7	Antihistaminic	Histaminergic H1 receptor (Histamine?)	antagonist	Yes
**Astemizole**	68844-77-9	Antihistaminic	Histaminergic H1 receptor (Histamine?)	antagonist	Yes
**Clevidipine**	167221-71-8	Antihypertensive	dihydropyridine calcium channel	antagonist	Yes
**Amlodipine**	88150-42-9	Antihypertensive	Voltage-gated L-type Ca^2+^ channel	antagonist	Yes
**Carbimazole**	22232-54-8	Antihyperthyroid	Thyroid peroxidase	inhibitor	Yes
**Propylthiouracil**	51-52-5	Antihyperthyroid	Thyroid peroxidase	inhibitor	Yes
**Cholecalciferol**	67-97-0	Antihypoparathyroid	Vitamin D3	N/A	No
**Halofantrine hydrochloride**	36167-63-2	Antimalarial	Ferriprotoporphyrin IX	antagonist	Yes
**Anastrozole**	120511-73-1	Antineoplastic	Cytochrome P450 19A1	inhibitor	Yes
**Procarbazine hydrochloride**	366-70-1	Antineoplastic	DNA alkylating	activator	-
**Etoposide**	33419-42-0	Antineoplastic	DNA topoisomerase II	inhibitor	Yes
**Thioguanosine**	85-31-4	Antineoplastic	Hypoxanthine-guanine phosphoribosyl transferase	inhibitor	No
**Ropinirole hydrochloride**	91374-20-8	Antiparkinsonian	Dopamine D2 receptor	agonist	Yes
**Prasugrel**	150322-43-3	Antiplatelet	Purinergic receptor P2Y12	antagonist	Yes
**Piperidolate hydrochloride**	129-77-1	Antispastic	Cholinergic receptors	antagonist	Yes
**Ethaverine hydrochloride**	985-13-7	Antispastic	Voltage-gated L-type calcium channel	blocker	Yes
**Fenspiride hydrochloride**	5053-08-7	Antitussive	Bradykinin receptor	antagonist	No
**Famotidine**	76824-35-6	Antiulcer	Histamine H2 receptor	antagonist	Yes
**Amantadine**	768-94-5	Antiviral	Glutamate [NMDA] receptor	antagonist	Yes
**Aminophylline**	317-34-0	Bronchodilator	Phosphodiesterase 3, 4	inhibitor	Yes
**Zardaverine**	101975-10-4	Bronchodilator	Phosphodiesterase 3, 4D	inhibitor	Yes
**Dexrazoxane**	24584-09-6	Chemoprotectant	DNA topoisomerase II	inhibitor	Yes
**Iodipamide**	606-17-7	Contrastant	Cholesteryl ester transfer protein	inhibitor	No
**Tripamide**	73803-48-2	Diuretic	Sodium-(potassium)-chloride cotransporter	inhibitor	Yes
**Ciprofibrate**	52214-84-3	Hypocholesterolemic	Peroxisome proliferator-activated alpha receptor	agonist	Yes
**Teriflunomide**	163451-81-8	Immunomodulator	Dihydroorotate dehydrogenase	inhibitor	Yes
**Bethanechol chloride**	590-63-6	Muscle relaxant	Muscarinic acetylcholine receptor M1, M2, M3, M4	agonist	Yes
**Ifenprodil tartrate**	23210-58-4	Vasodilator	Adrenergic receptor Alpha-1	antagonist	Yes
**Betahistine mesylate**	54856-23-4	Vasodilator	Histamine H3 receptor	agonist	Yes

-, unknown target.

## Data Availability

The original contributions presented in this study are included in the article/Supplementary Materials. Further inquiries can be directed to the corresponding author.

## Supplementary Materials

The following supporting information can be downloaded at https://www.mdpi.com/article/10.3390/ijms27188367/s1.
