# Supplementary material for: An Unbiased Drug Screen in a Drosophila Model of LMNA-Muscular Dystrophy Identifies Calcium Channel Blockers as Potential Treatments
Source: Int J Mol Sci. 2026 Sep 19;27(18):8367. doi: 10.3390/ijms27188367 (PMC13607455; doi:10.3390/ijms27188367)
Supplement: Supplementary file 1 [file ijms-27-08367-s001.zip › ijms-4400793-supplementary/ijmsSupplementalFINAL.pdf]

Table S1: A list of the drugs used in the primary screen.

Table S2: Purchasing information for compounds used in secondary drug screen.

Compound	Source	Catalog Number	Lot number
Clevidipine butyrate	Sigma Aldrich	SML-2607	0000362362
Amlodipine besylate	Sigma Aldrich	A5656	0000391896
Diltiazem hydrochloride	MedChemExpress	HY-14656	13070
Verapamil hydrochloride	MedChemExpress	HY-A0064	268925

Table S3: Expression of *LamC* S72P or H545P results in complete pupal lethality.

Mutant lamin	Live adults	Dead Pupae	Percent adult viability
S72P	0	695	0
H545P	0	359	0
M553R	0	480	0

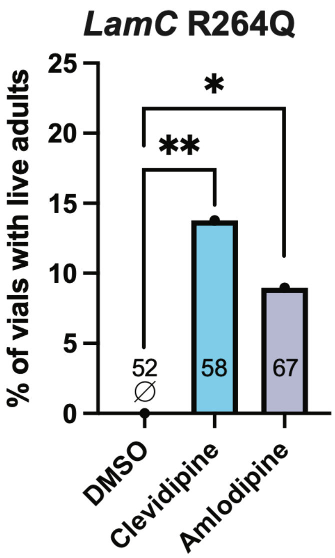

Figure S1. The secondary screen confirmed that the DHP-LLTCs clevidipine and amlodipine rescue lethality caused by *LamC* R264Q. The total number of vials scored is indicated. The percent of vials with live adults was plotted and significance determined using a one-way ANOVA with Dunnett's correction (GraphPad Prism).

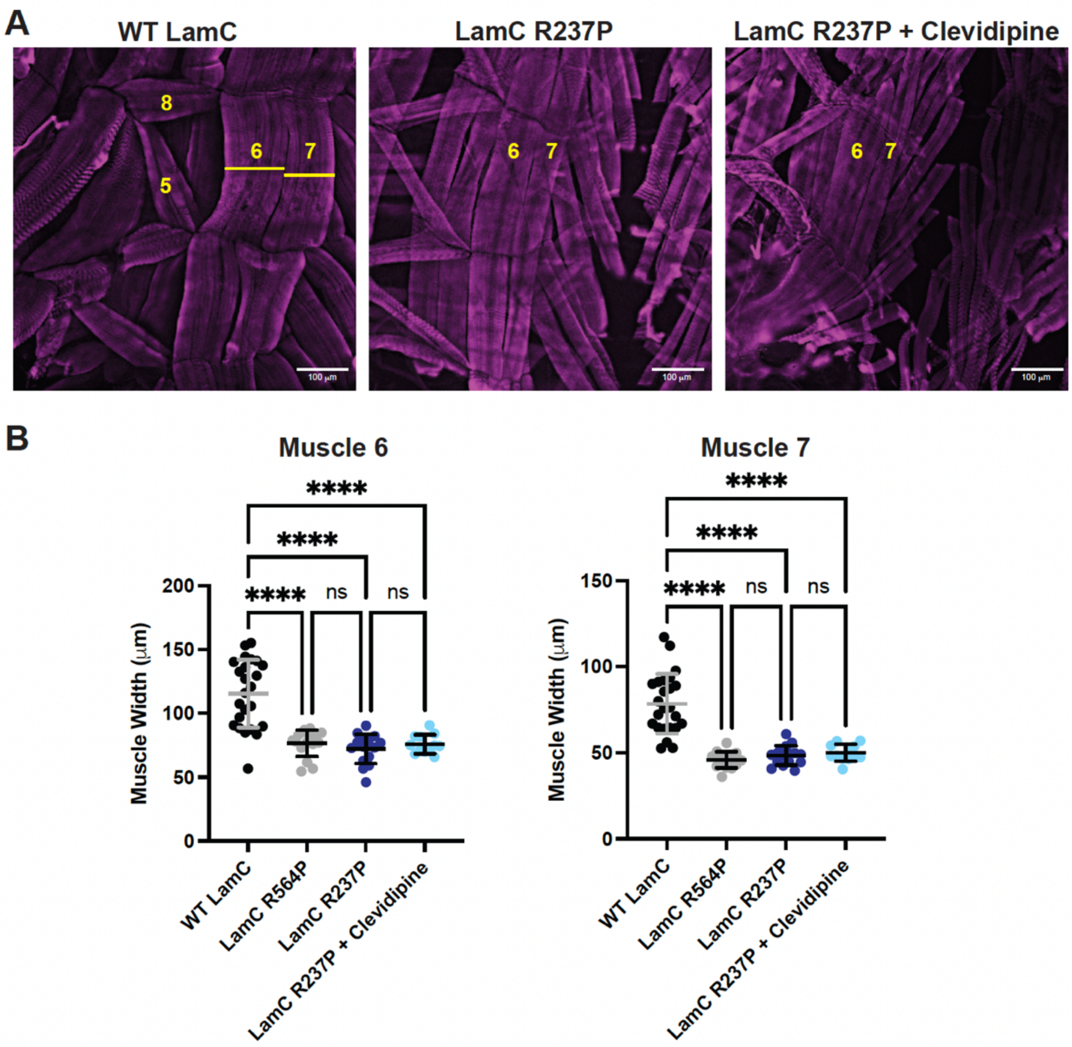

Figure S2. Clevidipine does not rescue the reduced muscle size caused by *LamC* R237P. A) The muscle size decrease due to expression of mutant *LamC* is not suppressed by clevidipine treatment. Larval body wall muscles were dissected, fixed, and stained with phalloidin (magenta) and DAPI (blue). B) The reduced size of muscles 6 and 7 in larvae expressing *LamC* R237P was not suppressed by clevidipine treatment. The width of muscles 6 and 7 were measured for control larvae and larvae expressing *LamC* R237P, untreated and treated with 0.5 μM clevidipine. Larvae expressing wild-type *LamC* served as a control. In addition, larvae expressing *LamC* R564P served as a positive control for reduced muscle size. The measurements showed that expression of *LamC* R237P caused reduced muscle width relative to larvae expressing wild-type *LamC*. The reduced muscle width was comparable to muscles of larvae expressing *LamC* R564P and clevidipine treatment did not rescue the reduced muscle size. Muscle fillets were prepared from three to six larvae per genotype and four measurements were taken from each muscle fillet. Error

bars represent standard deviation. Comparisons were performed using a one-way ANOVA with Dunnett's correction using GraphPad Prism.
